# Supplementary material for: Metabolomics Analysis Reveals the Effect of Two Alpine Foliar Diseases on the Non-Volatile and Volatile Metabolites of Tea
Source: Foods. 2023 Apr 7;12(8):1568. doi: 10.3390/foods12081568 (PMC10137691; doi:10.3390/foods12081568)
Supplement: Supplementary file 1 [file foods-12-01568-s001.zip › foods-2286523-supplementary.pdf]

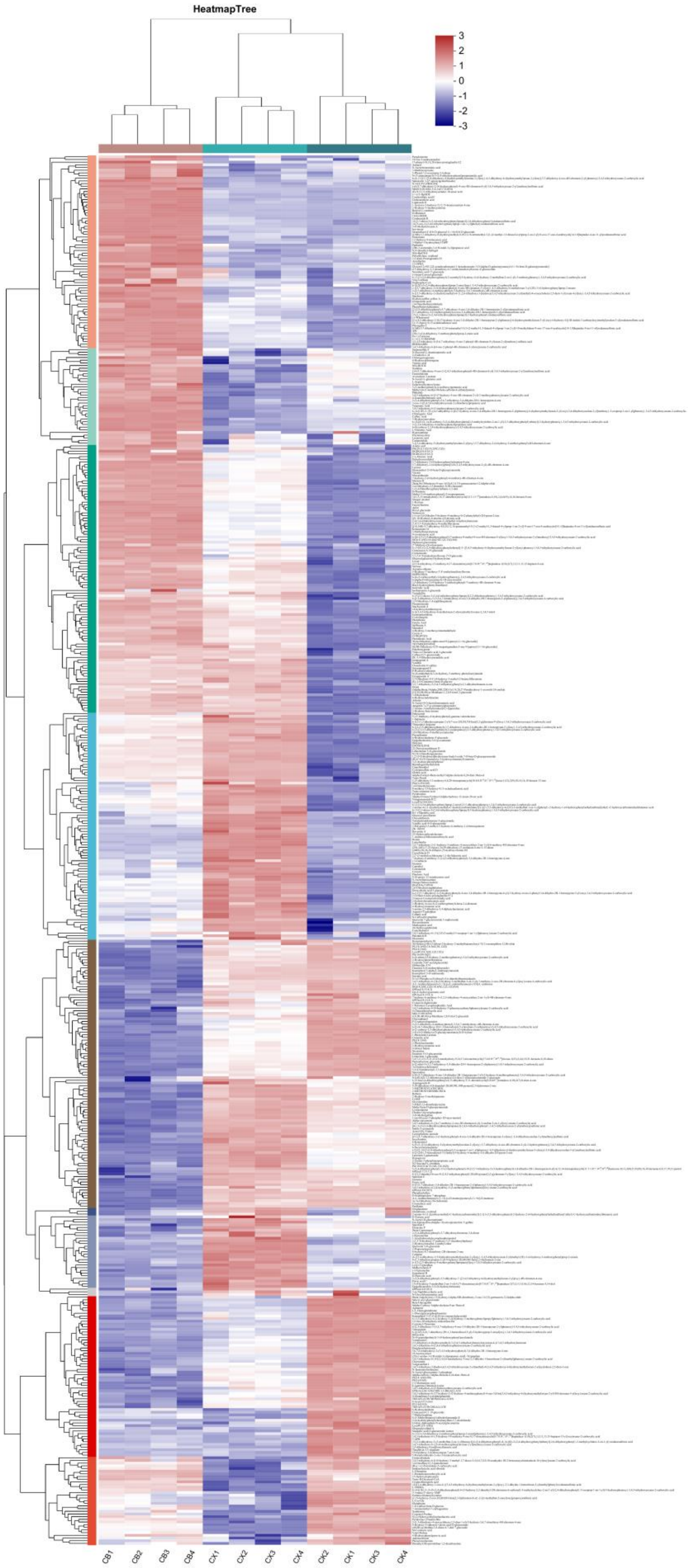
HeatmapTree
3
2
1
0
-1
-2
-3
CB1
CB2
CB3
CB4
CX1
CX2
CX3
CX4
CK2
CK1
CK3
CK4

Figure S1. A heatmap showing the relative variation of differential metabolites in HT, SS and BB. HT: Healthy tea shoots, SS: Tea shoots infected with small leaf spot disease, BB: Tea shoots infected with blister blight disease.

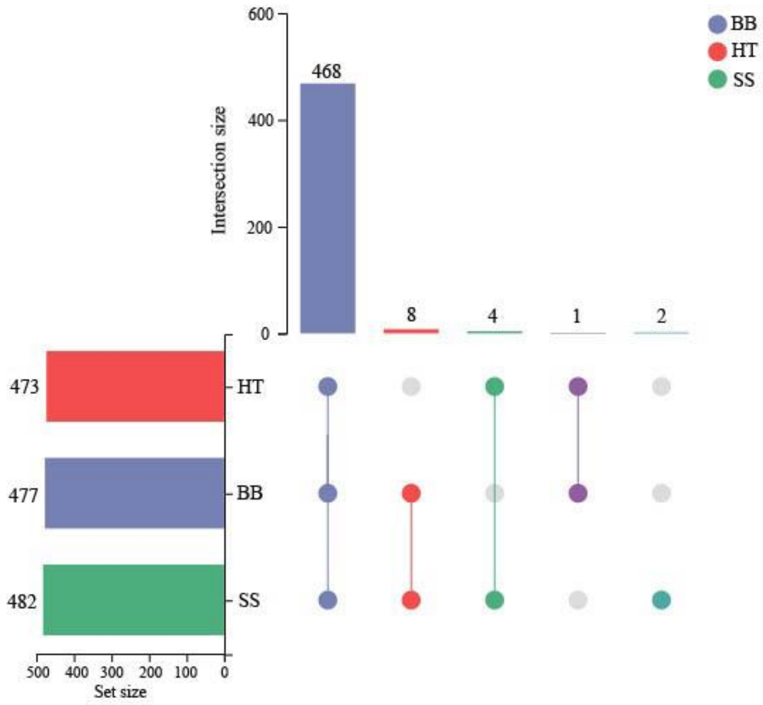

Figure S2. Overlapping relationship of differential metabolites among different samples shown in Venn diagrams in negative (-) ion mode. HT: Healthy tea shoots, SS: Tea shoots infected with small leaf spot disease, BB: Tea shoots infected with blister blight disease.
